# Feasibility and improvement of a three-dimensional printed navigation template for modified cortical bone trajectory screw placement in the lumbar spine

**DOI:** 10.3389/fsurg.2022.1028276

**Published:** 2022-11-02

**Authors:** Wenjie Shi, Mijiti Aini, Limin Dang, Alafate Kahaer, Zhihao Zhou, Yixi Wang, Abulikemu Maimaiti, Shuiquan Wang, Hailong Guo, Paerhati Rexiti

**Affiliations:** ^1^ Xinjiang Uygur Autonomous Region, Xinjiang Medical University, Urumqi, China; ^2^ Department of Orthopedics Second People's Hospital Kashgar District, Xinjiang Uygur Autonomous Region, Kashgar City, China; ^3^ Department of Spine Surgery, The First Affiliated Hospital of Xinjiang Medical University, Xinjiang Uygur Autonomous Region, Urumqi, China; ^4^ Department of Anatomy, College of Basic Medicine, Xinjiang Medical University, Xinjiang Uygur Autonomous Region, Urumqi, China

**Keywords:** spine implants, lumbar vertebra, 3D printing, reverse engineering, cortical bone trajectory, 3D navigation template

## Abstract

**Objectives:**

Compared with traditional pedicle screw trajectory, cortical bone trajectory (CBT) increases the contact surface between the screw and cortical bone where the screw is surrounded by dense cortical bone, which does not deform remarkably due to degeneration. We aimed to provide detailed information about the improvement of three-dimensional (3D)-printed navigation templates for modified CBT screw placement in the lumbar spine and evaluate the safety and accuracy thereof.

**Methods:**

Four human cadaveric lumbar spine specimens were selected. After CT scanning data were reconstructed to 3D models, either the left or right side of each specimen was randomly selected to establish a 3D-navigation template, mutually complemented with the surface anatomical structure of the lateral margin of the lumbar isthmus, vertebral plate, and spinous process. The corresponding 3D centrum was printed according to the CT scanning data, and a navigation template of supporting design was made according to modified cortical bone technique. The same template was used to insert CBT screws into 3D printed and cadaveric specimens. After the screws were inserted, the screw path of the 3D printed specimens was directly observed, and that of the anatomical specimens was scanned by CT, to determine the position and direction of the screws to analyze the success rate of screw placement.

**Results:**

Twenty cortical bone screws were placed in each of the 3D printed and anatomical specimens, with excellent rates of screw placement of 100% and 95%, respectively.

**Conclusions:**

We report the easy, safe, accurate, and reliable use of a 3D-printed navigation template to carry out screw placement by modified cortical bone technique in the lumbar spine.

## Introduction

Cortical bone trajectory (CBT) was a new lumbar screw trajectory proposed by Santoni in 2009 (1). Compared with the traditional pedicle screw trajectory, CBT increases the contact surface between the screw and cortical bone where the screw is surrounded by dense cortical bone (2–5) which does not deform remarkably due to degeneration (6, 7). CBT screws were predominantly designed for patients with osteoporosis (8, 9), and provide a new minimally invasive fixation option for lumbar and revision surgery, which has value in orthopedic clinics (9, 10).

The current traditional CBT technique still has imperfections reported in previous clinical, imaging, and anatomical studies (11, 12). To make up for any deficiencies, and to further increase the strength of screw placement, we altered and modified the insertion point and track of CBT. Without changing the horizontal axis, the vertical axis of the insertion point of the cortical bone screw was moved from the conventional mid-perpendicular line of the articular process (the 5 o'clock orientation in the left pedicle and the 7 o'clock orientation in the right) (3) to the tangent line of the median wall of the pedicle (11, 13). Therefore, compared to traditional CBT, the insertion point of modified lumbar cortical bone screw placement tends to be more medial, which may easily perforate the medial side of the pedicle into the vertebral canal, posing a higher potential risk of nerve injury.

Recently, 3D printing techniques have been applied in orthopedic clinics (14, 15), showing significant advantages in the treatment of spinal diseases and providing an innovative method to improve accuracy in complex spinal surgical operations (16). We aimed to explore the safety and veracity of 3D printed navigation templates to assist modified CBT (MCBT) screw placement, as well as investigate the auxiliary operating skill and navigation template performance improvement, so as to provide some fundamental basis for further clinical application.

## Materials and methods

### Date and location

The experiments were completed in the Anatomy Teaching-Research Office and the Department of Spine Surgery at the Orthopedic Center of Xinjiang Medical University from April 2019 to June 2022.

### Specimens

**Figure 1:**
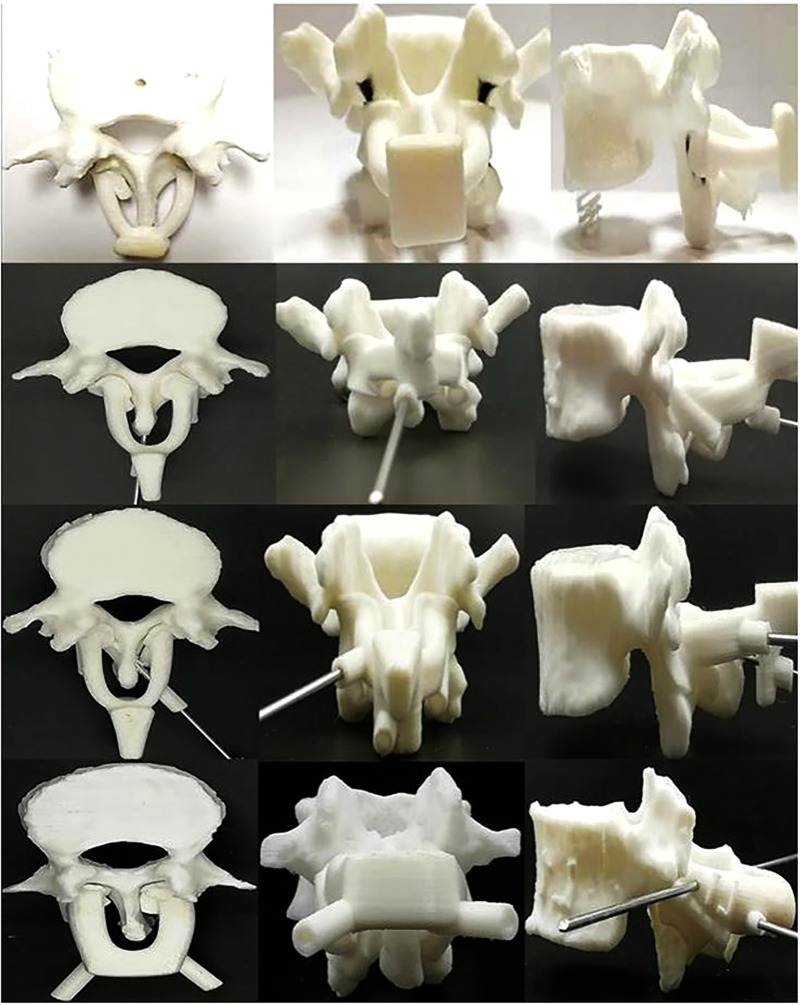
3D-printed lumbar spine specimens and four generations of navigation templates. CT scans were performed on four anatomical specimens. After reconstructing the data in 3D, each complete lumbar model was inputted into a 3D printer, and the 3D vertebral specimens were printed with anatomical specimens at a ratio of 1:1 for experimental study. During the test, we continuously optimized and upgraded the navigation templates. From the first generation (top) to the fourth generation (bottom) navigation templates, the performance of the template, including adhesion, stability, convenience, safety, and other aspects, continued to improve.

Four intact human wet cadaver lumbar specimens (two males and two females), aged 61–77 years (average: 71 years), were provided by the Anatomy Teaching-Research Office of Xinjiang Medical University (Figure 1, 14). These specimens were pretreated with 10% (volume fraction) formaldehyde solution, and were confirmed to be free of lumbar fractures, tumors, tuberculosis, and malformation by x-ray. The L5 vertebra of Specimen 3 had isthmus breakage and spondylolisthesis.

### Methods

#### Design of guide hole of cortical bone screw navigation template

**Figure 2:**
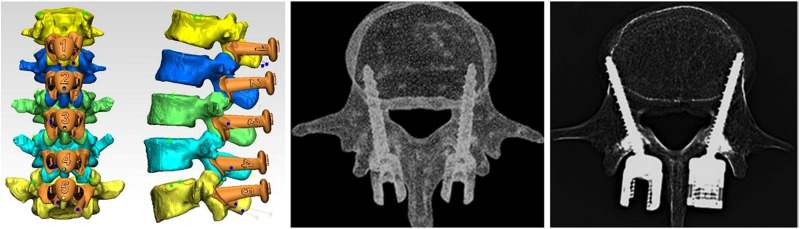
Difference between the traditional CBT and modified CBT technique, and the shape design of the 3D-navigation-template matched with different vertebra (middle picture reference: (25). To make up for the deficiency and further increase the strength of screw placement, we altered and modified the insertion point and track of CBT. Without changing the horizontal axis, the vertical axis of the insertion point of the cortical bone screw was moved from the conventional mid-perpendicular line of the articular process to the tangent line of the median wall of the pedicle (11).

High-resolution computed tomography (CT) data (AQUIRRON 16, Philips, Amsterdam, Netherlands) was performed on the four lumbar specimens. In Mimics 19 (Materialize, Leuven, Belgium), the CT scanned original data (DICOM format) underwent a reverse reconstruction operation. First, the coronary section at the midpoint of the long axis of each lumbar pedicle was taken as a hypothetical dial, to select the position of screw placement (Figure 2). In our application of the proposed modified method, the horizontal axis of the screw placement point was at the same level with the 6 o’clock orientation of the dial, the vertical axis was more medial at the tangent line of 3-o'clock orientation of the hypothetical dial, and the intersection point of the two axes was set as the origin of the Z-axis (17). The entry point was directly behind the Z-axis to the surface of the vertebral plate, with the corresponding projection position determined and set as the real entry point of screw, while the screw exit point was at the cortex of limbic bone of the end plate of the vertebra. A line was sketched on the above-mentioned established reference plane (ideal screw trajectory line), used by the computer to simulate the design of a 4.5-mm secure channel (screw trajectory diameter) between the screw entry and exit points, so as to generate an ideal screw trajectory. The selected 4.5 mm diameter resulted from the fact that Chinese people are smaller and that dry specimens or wet anatomical specimens soaked in formalin are fragile compared to normal living human bone. Because the modified screw entry point is closer to or more medial than the midline, the screw holds a tighter trajectory with the medial wall of the pedicle.

The lumbar segment was split layer by layer, and single vertebra data (STL format) were imported into Geomagic Studio (Raindrop, North Carolina, USA) for model repair and then into Z Brush for model reconstruction and optimization with the DynaMesh tool. After exporting the OBJ document, the data were imported into MAYA software (Autodest, San Rafael, California, USA) using the polygons module for the design. The designed pedicle implanted screw trajectory and Kirschner wire fixation hole were produced and performed, the split model set was exported as an OBJ document, and imported into Geomagic Studio. The final navigation template pattern was obtained and finally imported into MIMICS 19 to perform a Boolean calculation with the vertebra, under a tolerance of 0.3 mm, to complete the design of the guide hole of the navigation template screw.

#### Design and manufacture of contact surface between navigation template and specimens

In the Matic-3 STL 10 software (Materialize, Leuven, Belgium), the Wave Brush Mark tool was used to extract the anatomy data required for the margin of the spinous process basilar part, lateral margin of the lumbar isthmus, and vertebral plate superficial structure. This was skewed by 2.0 mm, and the model was exported from STL to Geomagic Studio. Simultaneously, the screw trajectory model, navigation template grip, and Kirschner wire fixation hole manufactured by MAYA were imported to perform the Boolean calculation. Finally, the screw trajectory of the navigation template and the trimmed boundary were connected, and the design and manufacture of the navigation template was complete.

#### Preparation of the specimens for screw placement

A CT scan was performed on the four anatomy specimens. After 3D reconstruction of data in Mimics 19, each intact lumbar vertebra model was inputted into a 3D printer, MBot Grid2 (Zheng Tian Medical Device, Tianjin, China), to print in 1:1 proportion with anatomical specimens. This was then attached to the 3D-printed navigation template, so as to facilitate follow-up improvement and the experimental operation of screw placement.

#### Screw placement

All screw placements in specimens were performed by spine surgeons without any experience of lumbar cortical bone screw placement. One side of each specimen was randomly selected to place each screw. To avoid wasting the specimens, a preliminary experiment was performed on the 3D printed vertebra. The navigation template was optimized and updated and, once confirmed to be accurate, safe, and reliable, the final test was performed on the anatomical specimens.

The navigation template screw placement processes on the 3D printed vertebra and anatomical specimens were as follows. First, the navigation template, with the isthmus lateral margin, vertebral plate, and spinous process as anatomic landmarks, was attached to the corresponding vertebra. The fit between the navigation template and the above-mentioned skeletal anatomical structure of the related vertebra was confirmed. We initially found that, when using a drill or Kirschner wire to drill through the guide hole of the navigation template, the template shook significantly during the rotation, whether held by the surgeon alone or with an assistant. This may have been caused by the small volume and weight of the navigation template itself and the small area held by the operator and may affect the accuracy of cortical bone screw placement. To solve this, a Kirschner wire was used to temporarily fix the navigation template, the attachment carefully checked, and a 2.7-mm drill used to trepan prior to screw placement. We drilled with an electric drill along the guide hole, to a depth of 35.0/40.0 mm, depending on the screw length. A 4.5-mm screw tap was used to extend the screw trajectory, and a probe was used to gently confirm the screw trajectory. Once the walls of the hole were confirmed to be smooth and continuous, a 4.5 × 35.0/40.0 mm titanium alloy lumbar back cortical bone screw was inserted.

In total, 40 specific cortical bone screws were placed in the corresponding specimen, with 20 placed in the 3D printed vertebra and the remaining 20 placed in human anatomical specimens.

### Evaluation criterion for screw placement

Due to the absence of current criteria for evaluation of CBT screw placement, a less than ideal alternative was adopted. The evaluation criterion was scored as previously described (18): grade I, the whole screw was inserted in the pedicle (excellent); grade II, less than 50% of the pedicle screw diameter penetrated the pedicle; grade III, more than 50% of the screw diameter penetrated the pedicle. Grade II and III were considered as negative screw positions.

### Main observation index

After the completion of screw placement, a CT scan was repeated under the same conditions. The scan was imported into Mimics 15.01 to evaluate the success rate of lumbar posterior cortical bone screw placement.

### Statistical analysis

We choose the random matching design, because the statistical efficiency of this design will be higher, hoping to make up for the small sample size of this study. The specific measures are as follows: the order of anatomical specimens is determined by simple random sampling one by one without putting back by drawing lots (The experimental serial number of each anatomical specimen is randomly selected on the drawing strips with numbers from 1 to 4 respectively). After sorting, the anatomical specimens are selected in order, and two numbers are simply and randomly selected from 0 to 100.If the first number is greater than the second number, we use the modified CBT method to place the nail on the left side of the corpse. If the first number is less than the second, the right side of each corpse is fixed with the modified CBT method. Follow this method until the completion of all four specimens of one side screw implantation. The data were collected and analyzed with SPSS 17.0 (IBM, New York, USA). Statistical analyses (chi-square test) were used to evaluate success rates of screw placement among groups. The test criterion was *α* = 0.05 on both sides, and *P* < 0.05 indicated a statistical significance.

### Human and animal rights statement

Approval was obtained from the ethics committee of the Anatomy Teaching-Research Office of Xinjiang Medical University. The procedures used in this study adhere to the tenets of the Declaration of Helsinki.

## Results

**Figure 3:**
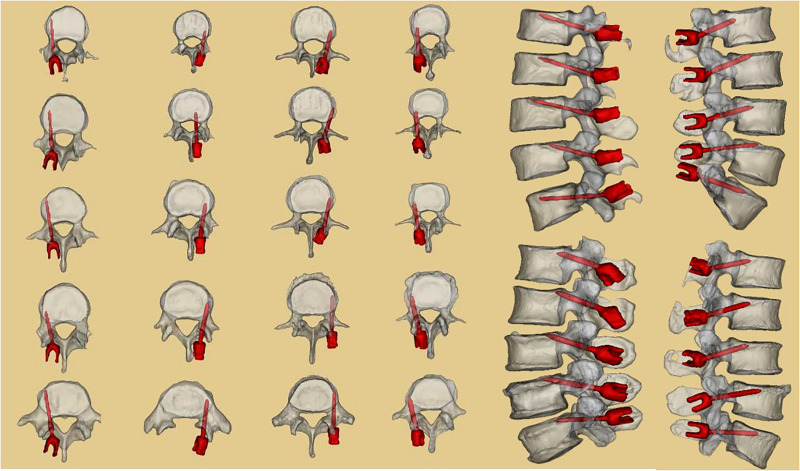
CT scan results of the four human anatomical specimens after screw placement. Twenty screws were placed in the four anatomical specimens under the aid of 3D-printed navigation templates. CT scans and three reconstructions were performed to observe screw placement. One screw was placed with an undesirable outcome.

When the screws were inserted into the printed and anatomical specimens with the aid of the 3D printed navigation template, we observed good process and stability of the navigation template. By visual inspection, all 20 screws placed in the 3D printed template were grade I, with a favorable rate of 100%. Of the 20 screws placed in the human anatomical specimens, the CT scan results showed that all except one screw placed on the right of L2 of one specimen were completely placed inside vertebral canal. This screw was evaluated as grade III; all other screws were evaluated as grade I, with an excellent rate of 95% (Figure 3). The rate of the 40 screw placements was, therefore, excellent at 97.5%. From the statistical analysis, the screw placement results of the two groups was the same, so the difference between the success rates was not statistically significant (*P* > 0.05).

## Discussion

Twenty cortical bone screws were placed in each of the 3D printed and anatomical specimens, with excellent rates of placement of 100% and 95%, respectively. Our 3D-printed navigation template successfully aided easy, safe, accurate, and reliable screw placement.

At present, the clinical application of 3D-printing techniques mainly involve the manufacture of a physical model, printing of surgical auxiliary materials, or printing of implants (8, 19–21). In 3D screw navigation template techniques, digital DICOM data is provided by 3D CT for high-precision reconstruction and then is 3D edited according to the design of the surgeon; the designed navigation template is 3D displayed on the computer; and finally, it is 3D printed by the related equipment, and this individualized navigation template is applied during spine surgery. Under serious hyperplasia, malformation, and other unclear anatomical structures, using 3D spine screw placement navigation templates can significantly improve operation accuracy and safety, and reduce x-ray times, radiation dosage, and operation duration. Thus, to more accurately place modified cortical bone screws, we made use of current 3D printing techniques to design supporting navigation templates to assist screw placement, and continuously optimized and updated the navigation template. A navigation template was used to guide screw placement tests on 3D printing and anatomical specimens. After 40 screw placements in two groups, all except one screw on one human anatomical specimen were completely placed inside the vertebral canal; none of the remaining screws were found broken in the inner and outside arm of the pedicle. Although the placement of screws using the 3D template encountered challenges, the operation, performed by junior spine surgeons at medical university without any related experience (22), required only half or even less time than that of experienced clinicians with the assistance of x-ray, suggesting great potential practical application value in clinical settings.

Compared to traditional pedicle screw techniques, the insertion point of the cortical bone screw technique is closer to the spinal canal, so it requires higher screw placement skill of the clinicians, and surgical staff and patients both receive larger doses of x-ray radiation (23). performed single segment cortical bone screw inner fixation on 12 lumbar spondylolisthesis patients and placed 48 screws in total, among which four (8.3%) perforated the pedicle cortex and lumbar vertebra. We believe that successful placement of lumbar cortical bone screws using individual navigation templates is required to improve the stability of the navigation template and rationality of anatomical references, while a stable navigation template mainly depends on a well-designed template fit surface (24). Considered that the stability of the vertebra and spinous process as the navigation template fit surface is better than other guide templates designed with other bone landmarks. Our screw placement navigation template design is based on this idea. In addition to the original bone landmarks, the lateral border of the isthmus is also taken as a reference, forming an isthmus-vertebra-spinous process combined anatomical reference, which is conducive to improved attachment and stability of the navigation template and bone surface. Additionally, these anatomical markers generally do not produce obvious bone degeneration with aging.

A series of improvement measures were also taken in the design of the navigation template, which differ from other traditional cortical bone screw 3D placement navigation templates. First, to increase 3D navigation template stability in screw placement, we referred to other template designs, and added the contact area between the navigation template and vertebral plate bone surface, to enable improved attachment. Meanwhile, the lateral arms on both sides of the navigation template were connected through the top beam structure in the middle, which is also a method to increase the contact area between the navigation template and bone, increase the stability of the navigation template when drilling, and prevent offset. We also designed a wider holding platform on top of both lateral arms, allowing the surgeon to easily hold the template to place the screw. By applying downward force with the hands, the navigation template attached more closely and tightly with the vertebral plate surface, which is good for follow-up temporary fixation with a Kirschner wire. In all our screw placements, only one placed into the L2 spinal canal of an anatomical specimen was evaluated as grade III. A follow-up improvement will be to increase the guide hole integrity to cover at least 3/4 of the diameter; at least 270° around the sleeve and drill. In cases where the spinous process hinders the side wall of the guide hole, the length of the guide hole will need to be reduced as much as possible. Even if the guide hold is 5-mm short but still complete, with a stable work sleeve and follow-up screw placement, the screw can be inserted in the appropriate direction, avoiding placement error. If the spinous process is blocked, we consider thinning the bone on both sides of the spinous process to make the guide plate adhere to the bone surface to the greatest extent. If it is still ineffective, the bone of the lower 1/3 spinous process can be removed and the proximal bone can be retained as much as possible, so as to protect the attachment of the supraspinal ligament as much as possible and increase the local stability. Modern medicine is becoming more individualized, precise and digital. We can choose CBT or MCBT according to the the exact level or position of the lumbar spine and preoperative imaging data of patients, and even combine the two (14).

Second, due to the supraspinous ligament, we designed the left and right arms of the 3D navigation template and middle top beam in the form of an arch bridge. This may help the surgeon and assistant to observe when the Kirschner wire is inserted from both sides, preventing inserting the screw too deep or even penetration into the vertebra to cause a neurological function lesion. The arch shape design is also good for mechanical stability of the navigation template. Furthermore, we increased the thickness of the side arms of the navigation template, so that it remains stable when the Kirschner wire penetrates the spinous process from one side. In addition, the height of the arch can be designed according to the thickness of the supraspinous ligament, enabling the 3D navigation template to straddle the spinous process and the supraspinous ligament above it. This helps to maintain distance with the supraspinous ligament; thus, the bottom of the side arms of the template can be well attached to the surface of the vertebral plate, rather than suspended (22, 25).

Third, it was found that, due to the small size of the 3D navigation template, during intraoperative guiding, it was not convenient for the surgeon or assistant to hold the template for a long time, and the rotation of the drill may change its direction at any time. To solve this problem, we preserved two fine holes in the navigation template use a 2-mm Kirschner wire for temporary fixation. The original design, to use one Kirschner wire to fix the template on the spinous process, cannot provide a solid attachment between the template and the vertebral plate surface, and the navigation template still shakes during drill rotation. As improved with time, the final Kirschner wire fixation scheme adopted a cross placement method at two different planes (coronal plane and sagittal plane) to temporarily fix the 3D navigation template solidly at multiple planes. As we are operating through a small incision, the angle of the two crossed Kirschner wires in the coronal plane should be small as possible; if this angle is too large, it hinders placement of the wire. The ends of the Kirschner wire should cross properly in the sagittal plane, so that it may smoothly enter the middle of the side arms on both sides of the navigation template, increasing navigation template stability. We eventually improved the navigation template material and used transparent macromolecule polyethylene, so that the surgeon could observe the wire in detail, to prevent inserting it too deep.

**Figure 4:**
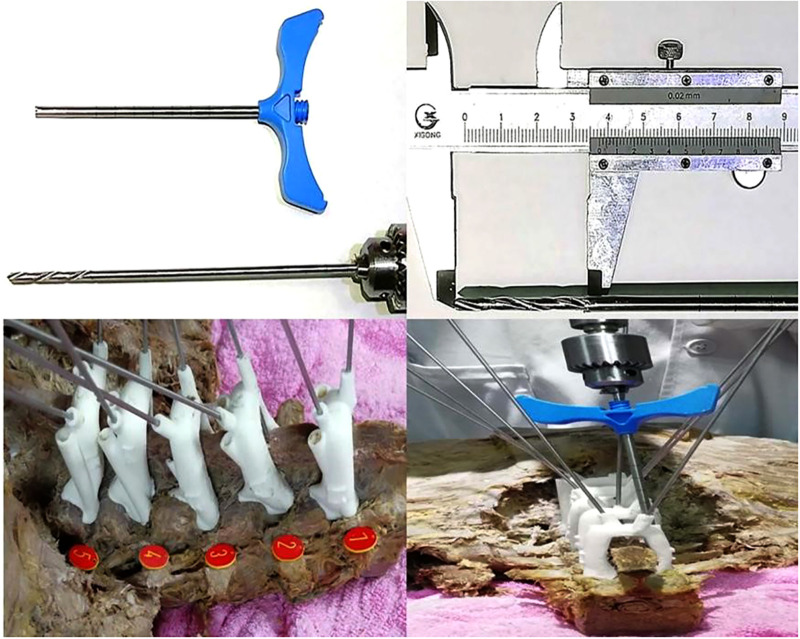
Improvement and specific application of inner core structure of the 3D-printed navigation template. We found that the working channel of the spinal balloon expandable bone cement tool can be used well with the cortical bone screw 3D printed navigation template, which increases the strength of the inner wall of the guiding hole, improves the accuracy of screw placement, and avoids breaking the inner wall and a large amount of debris during the drilling process.

Fourth, during practical guiding using the navigation template, we discovered that, because the navigation template is made from macromolecule polyethylene, the direction of drilling may deviate when the inner wall of the guide hole makes contact with the drill. This may also be caused by the hardness of the navigation template material, reducing navigation template accuracy. The only way to prevent this is to further increase the strength of the inner wall of the guide hole, making it strong enough to prevent drill deviation. We improved the inner wall structure of the guide hole in the navigation template by placing a stainless steel cannula used for limb fracture fixation inside it, and the problem of deviation was solved. This also solved the problem of the numerous chippings generated during drilling in the original inner wall of the navigation template. These chippings are likely to enter the vertebra of a patient during drilling, causing immunological reactions. In addition to the metal cannula, we found that a series of puncture tools used in the treatment of balloon dilation for spinal compression fractures can combine with the design of this navigation template, to make screw placement safer and more reliable (Figure 4).

**Figure 5:**
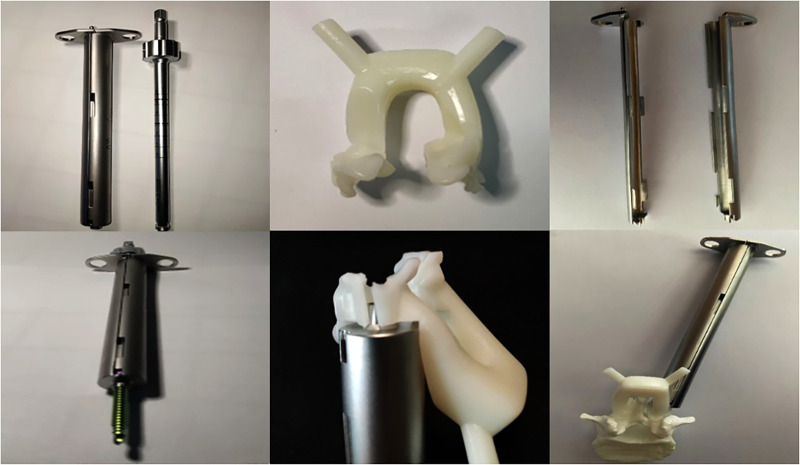
Removable minimally invasive 3D navigation system. The system provides a set of tools for the inner wall of metal for the nail setting channel, including the split metal sleeve combined with the long-rod metal screwdriver, supporting the fifth generation of 3D guide plates to establish and extend the surgical channel. The screw can directly contact the bone interface through the surgical channel, reducing damage to blood vessels, nerves, and surrounding tissues, and reducing trauma. At the same time, the disassembly procedure is simple and fast.

However, the metal sleeve used in limb fractures and bone cement forming surgery has complicated surgical steps: the drill bit and wire are used to approach the opening in the vertebral body, the Kirschner wire and 3D navigation template are removed, and the screw inserted. A relatively simple solution is to use a hollow screw to implant the drill bit along the previous opening. In a previous study, a disposable 3D navigation template made of stainless steel or alloy was also considered, but was not appropriate because of its high production cost and long production time. Combined with the knowledge from previous studies, and the operation experience of screw placement, we designed a detachable screw placement tool that can provide the inner wall strength of metal and is suitable for minimally invasive implantation. By seamless connection with the guide hole of the fifth generation 3D navigation template, this ensures that the screw is inserted accurately in the direction of the guiding hole, with a metal-to-metal interface and without any debris (Figure 5). With the help of the power system, the screw is inserted to a certain depth and in a specific direction until stable. Then, the 3D navigation template and the minimally invasive screw placement sleeve are removed, and the screw tail thread of the last section is screwed by hand. In this way, the operator can better perceive the torque when screwing into the final stage of cortical bone, avoid splitting the CBT nail screw, and reduce the occurrence of complications. It is recommended that the current clinically used percutaneous minimally invasive placed hollow screw and supporting tools can be combined with this navigation template.

In summary, the cortical bone screw trajectory technique is a new lumbar posterior internal fixation technique, with improvements and a screw trajectory that differs from traditional pedicle screws where the failure rate is always high and depends upon the manual screw placement experience of the surgeon. We designed a safe insertion angle, screw diameter, and other indexes through 3D reconstruction and reverse engineering techniques, and utilized a 3D printing technique, to verify the anatomical vertebra and navigation template. We proved that our technique makes it easier and safer for spine surgeons without any experience to place screws using a navigation template. In clinical practice, our 3D printed navigation template and special tools can further improve the accuracy and safety of modified cortical bone screw placement.

## Data Availability

The original contributions presented in the study are included in the article/Supplementary Material, further inquiries can be directed to the corresponding author/s.
